# A Dosimetric Comparison of Intensity-Modulated Proton Therapy, Volumetric-Modulated Arc Therapy, and 4π Non-Coplanar Intensity-Modulated Radiation Therapy for a Patient with Parameningeal Rhabdomyosarcoma

**DOI:** 10.7759/cureus.1673

**Published:** 2017-09-10

**Authors:** Tiffany W Chen, Julian Sison, Becky Lee, Arthur J Olch, Andrew Chang, Annelise Giebeler, Kenneth Wong

**Affiliations:** 1 Department of Radiation Oncology, UT Health San Antonio Cancer Center; 2 Robert Wood Johnson Medical School; 3 David Geffen School of Medicine, UCLA; 4 Department of Radiation Oncology, Keck School of Medicine of the University of Southern California, Los Angeles, CA; 5 Department of Radiation Oncology, Scripps Proton Therapy Center

**Keywords:** 4pi radiotherapy, intensity modulated proton therapy (impt), rhabdomyosarcoma, alveolar, parameningeal, pm-rms, volumetric modulated arc therapy (vmat), intensity-modulated radiotherapy (imrt), proton therapy

## Abstract

Rhabdomyosarcoma (RMS) is the most common soft tissue sarcoma in children and manifests as two major histological subtypes: embryonal and alveolar. The five-year local failure rate for RMS at parameningeal sites (middle ear, mastoid region, nasal cavity, etc.) is around 17% despite multiple Intergroup Rhabdomyosarcoma Study Group (IRS) trials conducted to determine the optimal radiation treatment regimen. This case report explores the use of intensity-modulated proton therapy (IMPT) for a 10-year-old child who presented with left eye irritation, facial pain, and headaches and was found to have an alveolar parameningeal rhabdomyosarcoma. He received systemic therapy as well as radiation therapy to 5,640 cGy and 4,320 cGy over 24 fractions, prescribed for gross tumor extension and adjacent high-risk involved sites, respectively, via simultaneous integrated boost. Approximately two years following treatment, the patient has had no recurrence of his RMS with no distant metastases. In addition, his presenting symptom of left eye irritation has improved. His only side effect from radiation at this point is short stature, possibly due to growth hormone deficiency. The patient’s IMPT plan was compared with volumetric-modulated arc therapy (VMAT) and 4π non-coplanar intensity-modulated radiation therapy (IMRT) plans, and comparisons of isodose lines show decreased dose to the distal brain tissue with preserved target conformality by IMPT. IMPT also allowed for increased sparing of the patient's retina, lens, and lacrimal gland. All radiation plans achieved conformal dose coverage to the planning/scanning target volumes, while the IMPT plan is potentially better at sparing the patient from developing long-term optic apparatus side effects and neurocognitive defects. In this case, IMPT is comparable, if not favorable, when long-term side effects can be reduced while maintaining dose conformality and local control.

## Introduction

Rhabdomyosarcoma (RMS) predominates in children under six years of age and is the most common soft tissue sarcoma of childhood, with around 350 cases per year [1]. Prognosis and treatment of this disease are highly dependent on histology and staging. The two major histological subtypes for RMS are embryonal rhabdomyosarcoma (ERMS) and alveolar rhabdomyosarcoma (ARMS), with a poorer prognosis associated with the latter [2]. The Intergroup Rhabdomyosarcoma Study Group (IRS) has established staging and clinical grouping systems to help describe the extent of disease, and the Children’s Oncology Group has developed a risk stratification method based on these parameters [3]. The clinical group is determined by the extent of surgical removal and presence of metastases, whereas staging is determined by whether the location is favorable (orbit, eyelid, ear lobe) or unfavorable (parameningeal), tumor size, and the presence of metastases as well [3].

Low-risk RMS cases are usually not treated with radiotherapy, as possible radiation sequelae can include endocrine deficits, hearing loss, cataracts, and neurocognitive defects [1]. However, parameningeal rhabdomyosarcoma (PM-RMS) exhibits high local failure rates and one-year survival rates below 50% after relapse [4-5]. Complete surgical resection is often impossible, making radiotherapy necessary for local control. The goals of radiotherapy are to maximize the dose to the primary site with adequate margins to include adjacent meninges [3] while minimizing dose to nearby structures, such as the lens and optic nerve.

In recent decades, four IRS trials have evaluated various doses and fractions for RMS radiotherapy. Doses > 50 Gy (in comparison to 40 - 45 Gy) did not produce substantial improvements for PM-RMS patients in terms of five-year local failure rates, which have hovered around 17%. There was also no significant difference between conventionally fractionated (50.4 Gy in 28 daily fractions) and hyperfractionated (59.4 Gy in 54 twice-daily fractions) radiotherapy [3]. Other studies have evaluated IMRT for PM-RMS and have demonstrated similar local control outcomes [4].

The use of protons may reduce the late effects experienced by patients. One dosimetric study has demonstrated improved sparing by protons for most normal tissues and lower integral dose in comparison with IMRT for PM-RMS [6]. Two follow-up studies of patients treated with proton therapy resulted in diminished late effects and comparable local control to photons [1, 7]. Unfortunately, there has been little improvement in local control by any technique, and to our knowledge, few studies have evaluated the efficacy of IMPT for PM-RMS. We present the case of a patient with Group III, Stage 3, parameningeal ARMS treated with IMPT to highlight issues when considering various radiotherapy techniques for PM-RMS.

## Case presentation

**Figure 1:**
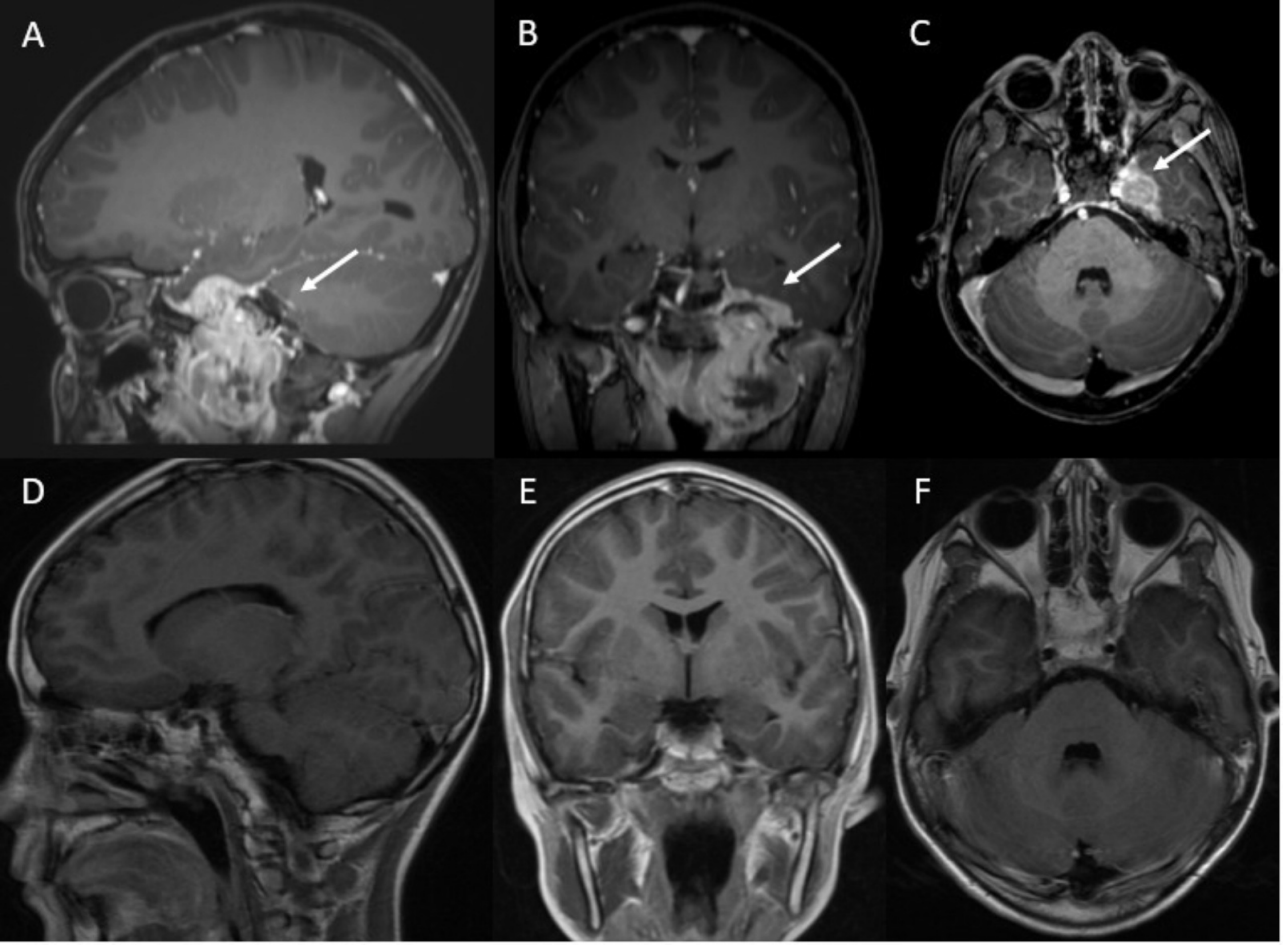
Parameningeal rhabdomyosarcoma in a 10-year-old male Sagittal (A), coronal (B), and axial (C) slices of T1-weighted MRI brain showing a hyperintense, enhancing lesion involving the left foramen ovale (blue arrows) pre-radiotherapy. Sagittal (D), coronal (E), and axial (F) slices of T1-weighted MRI brain showing no parenchymal signal abnormalities post-radiotherapy.

A 10-year-old male presented with left-sided facial pain and headaches and was initially thought to have a trigeminal schwannoma after a CT head scan revealed a 3.6 x 4.4 cm mass (Figure 1). Though the patient was scheduled for resection, interval pain prompted him to visit the emergency department, where magnetic resonance imaging (MRI) of the brain showed growth of the mass with left-sided involvement of the middle cranial fossa, cavernous sinus, Meckel’s cave, as well as mass effect into the nasal and oropharyngeal airway. Ophthalmology consult noted cranial nerve V1 dysfunction and left eye irritation with redness and discharge. The result of subsequent biopsy was consistent with Group III, Stage 3 parameningeal alveolar rhabdomyosarcoma (RMS) with fluorescence *in situ* hybridization (FISH) negative for FOXO1 and rearrangement of 13q14. Definite metastases were ruled out by a CT chest and bone scan.

The patient’s past medical history and past surgical history were noncontributory with no prior radiation treatment. His family oncologic history was significant for the patient’s father having a remote history of early stage melanoma managed surgically, as well as maternal great-grandparents who had pancreatic cancer in their ninth decades of life.

The patient was admitted for chemotherapy, and at the time of initial consult with radiation oncology, the patient had complete alopecia and mild fatigue from chemotherapy with no other abnormal findings on physical examination. Facial symptoms had reportedly improved on chemotherapy.

The patient was counseled regarding the risks and benefits of receiving proton therapy, and IMPT was planned. During planning, the brain, optic apparatus, and cochlea were identified as organs at risk (OAR). The patient underwent a dose-escalated regimen of 5,640 cGy and 4,320 cGy over 24 fractions, prescribed for gross tumor extension and adjacent high-risk involved sites, respectively, via simultaneous integrated boost (SIB). He tolerated radiation therapy well with the exception of some post-nasal drip, which was expected given the treatment sites involved. IMPT was completed after one month, and the patient finished chemotherapy five months later.

One year after finishing chemotherapy, the patient saw an endocrinologist for concerns about short stature and is being evaluated for growth hormone deficiency. He did not have diabetes insipidus or hypothyroidism. On the patient’s last follow-up visit two years after finishing treatment, he had no complaints and his left eye irritation had improved since the initial presentation. He was doing well in the sixth grade, maintaining a good appetite, and exercising regularly. The most recent MRI brain scan showed post-treatment changes with no recurrence of ARMS at the parameningeal site.

The patient’s IMPT plan was then compared to two other modern photon radiation therapy techniques, VMAT and 4π non-coplanar IMRT.

## Discussion

IMPT allows for both conformal treatment to the tumor tissue and sparing of surrounding OAR, which is especially important for pediatric patients because many survive to experience the late effects of radiation, such as visual or orbital complications, hearing loss, neurocognitive defects, and endocrine defects [1]. The IMPT plan for the presented case was designed with consideration of density and range uncertainties. A scanning target volume (STV) was created from the clinical target volume (CTV) by adding margins to account for setup uncertainty on the lateral aspect of the beam's eye view (BEV), as well as range uncertainty (accounting for 2.5% to 3.5% of the range). In addition, since rotational or positional offsets could lead to density changes in the mastoid/bone ratio of the beam path, a robustness evaluation of the dose volume histogram (DVH) was performed during plan evaluation.

**Figure 2:**
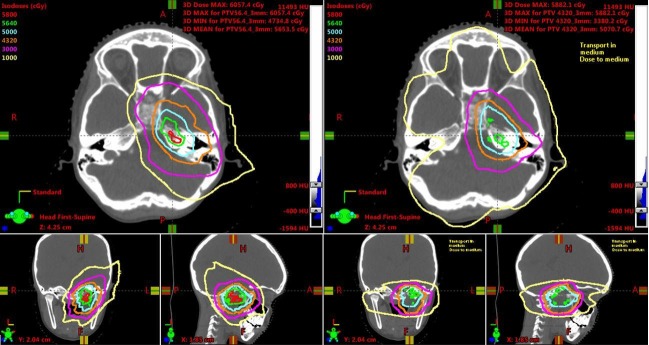
Isodose line comparisons between intensity-modulated proton therapy (left) and volumetric-modulated arc therapy plans (right). Isodose lines range from 1,000 cGy to 5,800 cGy, including 5,640 cGy (green) and 4,320 cGy (orange).

**Figure 3:**
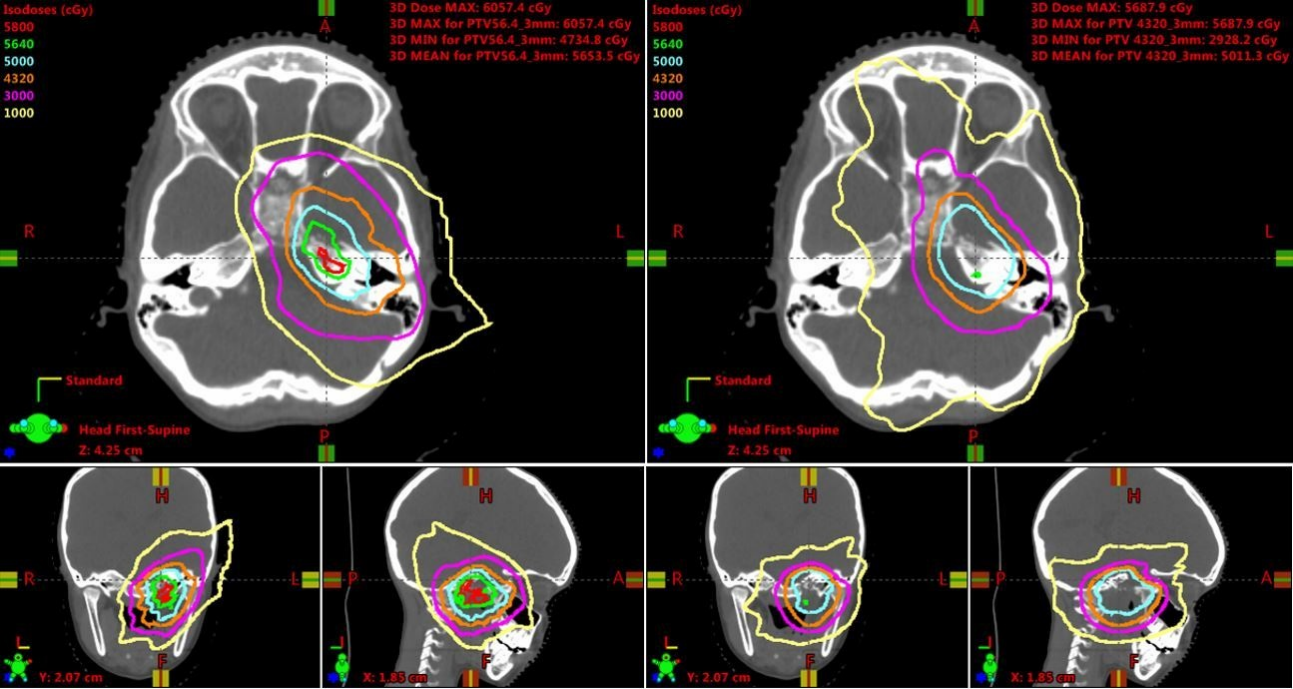
Isodose line comparisons between intensity-modulated proton therapy (left) and 4π plans (right) Isodose lines range from 1,000 cGy to 5,800 cGy, including 5,640 cGy (green) and 4,320 cGy (orange).

The IMPT plan was compared to VMAT and 4π plans designed for the same CTV. VMAT allows for one or multiple arcs to be used with simultaneous variation in gantry rotation speed, multileaf collimator (MLC) leaf positions, and dose rate, allowing for conformal treatment to be delivered in a faster time than competing radiation modalities, such as step-and-shoot IMRT. While VMAT has wide adoption and would be a widely available technique for RMS, 4π, a form of IMRT using noncoplanar beams that offer improved dose compactness, has been shown to further spare the surrounding critical structures without compromising target coverage in central nervous system tumors, such as glioblastoma [8]. The isodose lines show decreased dose to the distal brain tissue in the IMPT plan compared to the VMAT and 4π plans (Figures 2-3).

**Figure 4:**
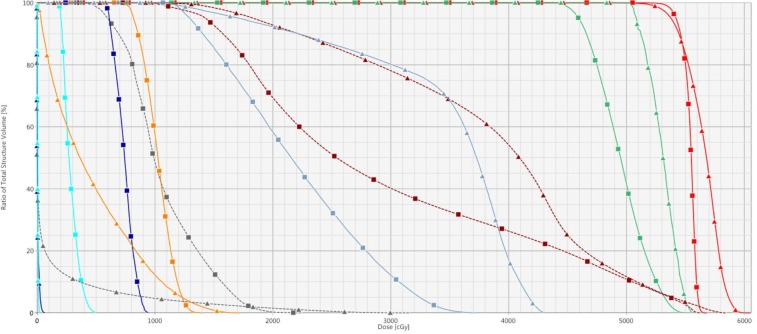
Dose volume histogram comparing intensity-modulated proton therapy (triangles) and 4π plans (squares) From right to left: the planning/scanning target volume (red), left cochlea (green), left temporal lobe (dashed dark red), left optic nerve (light blue), right temporal lobe (dashed grey), left retina (orange), left lacrimal gland (dark blue), and left lens (cyan).

Since the 4π isodose lines show improved sparing of OAR compared to the VMAT plan, only the DVHs for the 4π and IMPT plans will be discussed below. PTV coverage of the 4π plan and STV coverage of the IMPT plan were both excellent, with > 95% of the volumes receiving 95% of the targeted dose (Figure 4). Of note, the 4π plan was more homogenous with a lower maximal dose.

In the presented case, IMPT allowed for increased sparing of the patient’s optic apparatus on the side of his tumor, including the retina, lens, and lacrimal gland. The mean doses of these structures were lower than those from the 4π plan (Table 1). In addition, the maximum doses of the left lens and lacrimal gland were also significantly lower in the IMPT plan. In pediatric patients with PM-RMS, about 10% of long-term survivors will develop cataracts secondary to radiation treatment [9]; therefore, the upper limit of radiation to the lens has been reported to be around 5 Gy [6]. As seen in Table 1, the maximum dose to the left lens in the 4π plan is nearly 5 Gy, while the maximum dose in the IMPT plan is approximately 25 times lower. In addition, the lower integral dose to the brain by IMPT could potentially cause the patient to have decreased risk of developing neurocognitive defects than if 4π was used (Table 1).

**Table 1:** Comparison of Mean and Maximum Doses for Intensity-modulated Proton Therapy (IMPT) Versus 4π Plans IMPT: intensity-modulated proton therapy

Structure	IMPT Mean Dose (cGy)	4	π Mean Dose (cGy)	IMPT Maximum Dose (cGy)	4	π Maximum Dose (cGy)
Planning Target Volume	5,653.5	5,536.8	6,057.4	5,687.9
Left Retina	470.4	1,023.8	1,722.3	1,405.7
Left Lens	5.7	283.9	19.9	499.5
Left Lacrimal Gland	5.6	740.5	68.2	941.5
Left Temporal Lobe	3,845.8	2,974.4	5,852.0	5,624.9
Right Temporal Lobe	136.9	1,066.2	3,163.1	2,257.2
Pituitary Gland	3,385.8	1,664.2	4,401.1	4,001.6
Brain	364.0	563.9	5,747.6	5,605.6

The left temporal lobe fell in the region of the proximal dose plateau of the incoming proton beam from that direction, and the planning software used for the IMPT plan did not have a normal tissue optimization function for inverse proton planning. Therefore, the IMPT plan potentially could have been better optimized to lower the left temporal lobe dose. While the mean dose for the left temporal lobe was higher in the IMPT plan, the mean doses of the brain and right temporal lobe were lower than those in the 4π plan (Table 1).

In light of our current knowledge of the patient’s potential for growth hormone deficiency, the 4π plan was optimized to reduce the pituitary dose. Hence, there was greater sparing of the pituitary gland compared to the IMPT plan. In retrospect, it may have been possible to improve the original IMPT plan to further decrease the dose to the pituitary gland. Nevertheless, IMPT allowed for this patient to not only achieve local control but also have the potential reduction of risk of developing future radiation-induced visual and neurocognitive deficits, compared to using photons [10].

## Conclusions

Through IMPT’s greater sparing of the optic apparatus and brain compared to photons, the patient may be less likely to experience late effects of radiation, such as cataracts, chronic keratoconjunctivitis sicca, neurocognitive decline, and visual complications.
